# Real time noninvasive assessment of external trunk geometry during surgical correction of adolescent idiopathic scoliosis

**DOI:** 10.1186/1748-7161-4-5

**Published:** 2009-02-24

**Authors:** Luc Duong, Jean-Marc Mac-Thiong, Hubert Labelle

**Affiliations:** 1 Research Center, Hôpital Sainte-Justine, 3175 Côte-Sainte-Catherine, Montréal, Québec, H3T 1C5, Canada; 2 Department of Orthopaedic Surgery, Université de Montréal, PO Box 6128, Station Centre-ville, Montréal, H3C 3J7, Canada

## Abstract

**Background:**

The correction of trunk deformity is crucial in scoliosis surgery, especially for the patient's self-image. However, direct visualization of external scoliotic trunk deformity during surgical correction is difficult due to the covering draping sheets.

**Methods:**

An optoelectronic camera system with 10 passive markers is used to track the trunk geometry of 5 scoliotic patients during corrective surgery. The position of 10 anatomical landmarks and 5 trunk indices computed from the position of the passive markers are compared during and after instrumentation of the spine.

**Results:**

Internal validation of the accuracy of tracking was evaluated at 0.41 +/- 0.05 mm RMS. Intra operative tracking during surgical maneuvers shows improvement of the shoulder balance during and after correction of the spine. Improvement of the overall patient balance is observed. At last, a minor increase of the spinal length can be noticed.

**Conclusion:**

Tracking of the external geometry of the trunk during surgical correction is useful to monitor changes occurring under the sterile draping sheets. Moreover, this technique can used be used to reach the optimal configuration on the operating frame before proceeding to surgery. The current tracking technique was able to detect significant changes in trunk geometry caused by posterior instrumentation of the spine despite significant correction of the spinal curvature. It could therefore become relevant for computer-assisted guidance of surgical maneuvers when performing posterior instrumentation of the scoliotic spine, provide important insights during positioning of patients.

## Background

Adolescent idiopathic scoliosis (AIS) is characterized by a complex lateral shift of the spinal curve in the frontal plane, associated with a complex 3-D deformity of the trunk [1,2]. In some severe cases of scoliosis, posterior spinal instrumentation and fusion is performed to partially correct and prevent worsening of the deformity [3]. During this procedure, most of the surgical maneuvers are aimed at correcting the scoliotic spine and to ensure adequate spinal balance. However, scoliosis is also associated with an external trunk deformity, which has to be addressed during correction of scoliosis [4].

Direct intra-operative visualization of trunk deformity is difficult due to the draping sheets covering the whole trunk (including shoulders and pelvis). In particular, it is very difficult to evaluate precisely the shoulder, trunk and pelvic balance during surgery. Using intra-operative radiographs, Martin-Benlloch *et al. *have evaluated the lateral shift of C7 vertebra with respect to the sacrum and the shoulder asymmetry [5]. Although intra-operative radiographs are routinely used to verify position of the vertebral implants, they only provide limited information about the trunk geometry. Furthermore, they cannot be used for dynamic evaluation of the trunk deformity. Mac-Thiong *et al*. have reported the feasibility of intraoperative tracking of the trunk during scoliosis surgery using an electromagnetic motion capture device [6,7]. This technique was intended to be the first step towards computer-assisted guidance of surgical maneuvers in scoliosis surgery. Added to the fact that they only tested their technique on one patient, electromagnetic tracking involves many drawbacks. Ferromagnetic material present in the operating room (e.g. operating table, instruments, anesthetic equipment) can interfere with the tracking device and affects its accuracy [8-11]. In addition, the electromagnetic emitter can alter readings from electromyographic and anesthetic monitors. Also, the wiring used in electromagnetic tracking can be cumbersome in a surgical environment. Therefore, an optical camera system for intraoperative tracking of trunk geometry is introduced to overcome the technical limitations associated with electromagnetic tracking. The objective of this study is to evaluate the clinical relevance of an optical tracking technique in documenting the variation of the trunk geometry during scoliosis surgery.

## Methods

### Sample description

**Figure 1:**
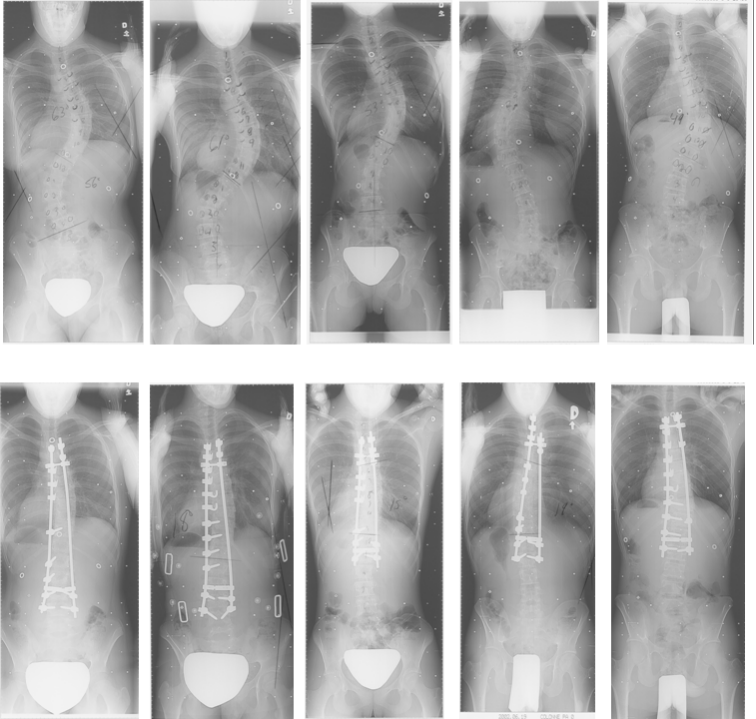
**Pre- and post-operative radiographs, presented respectively on top and bottom row**.

Five patients (2 males, 3 females) with AIS undergoing surgical correction of the spine by posterior instrumentation and fusion were recruited on a voluntary basis. Patients included in this study were all treated by posterior instrumentation and fusion, using a third generation instrumentation system. Two patients presented a double curve pattern (right thoracic, left lumbar) and two other patients were diagnosed with a single right thoracic curve. Finally the remaining patient showed a single left thoracic curve pattern. Preoperative Cobb angles, computed on standing radiographs ranged from 49 degrees to 63 degrees. Additional details on the patient characteristics considered for this study are described in Table 1. Pre- and post-operative radiographs are presented on Figure 1.

**Table 1:** Patient clinical characteristics, with Cobb angle measured during and after surgical correction of the spine.

	Patient 1	Patient 2	Patient 3	Patient 4	Patient 5
Age (years)	12	16	17	20	16
Sex	F	F	F	M	M
Curve pattern	Double right thoracic, left lumbar	Double right thoracic, left lumbar	Single, right Thoracic	Single, left Thoracic	Single, right Thoracic
Primary Cobb angle (degrees)					
Preoperative	63 (L: 56)	61 (L: 55)	53	57	49
Postoperative	21	18	15	19	22

### Acquisition protocol

**Figure 2:**
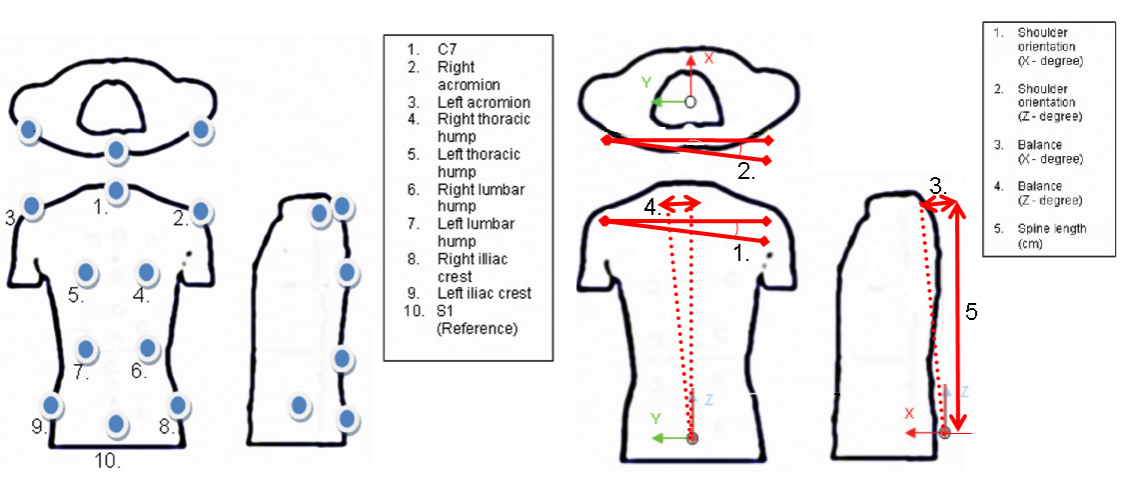
**A) Landmarks defined for trunk tracking, adapted from Mac-Thiong et al**. B) Angles defined for real-time tracking of the trunk external geometry.

In the operating room, ten specific anatomical landmarks are identified on the patient's trunk prior to sterile draping. These anatomical landmarks were adapted from the experiment on trunk tracking using electromagnetic sensors [6,7]. The landmarks used in this study are illustrated on Figure 2A and are defined as:

1) the spinous process of C7 vertebra,

2) the right acromion,

3) the left acromion,

4) the right thoracic prominence,

5) the left thoracic prominence,

6) the right lumbar prominence,

7) the left lumbar prominence,

8) the right superior posterior iliac crest,

9) the left superior posterior iliac crest, and

10) the spinous process of S1 vertebra.

The clinical indices computed for this study are adapted from a previous study done using a magnetic tracking system,[7] and are outlined on Figure 2B. Indices include the shoulder rotation (using landmark 2–3), the overall balance (using landmark 1–10), and finally the spine length (landmark 1–10). Rotation indices from shoulder were considered in the axial plane and in the frontal plane. Balance in the axial and frontal plane was computed.

The remaining landmarks (4–7) are gathered to provide a complete schematic representation of the trunk including the thoracic and the lumbar spine, and thus can be easily modified to visualize the effect of surgical maneuvers on the thoracic and lumbar hump in future studies.

Since the camera system is acquiring real-time data, respiratory motion of the patient during the acquisition might affect the accuracy of the indices computation. Landmarks located on the thoracic hump (4–5) were therefore used for visual gating, so to keep only 3-D data at the end of the respiratory cycle for indices computation.

### Optical tracking technique

**Figure 3:**
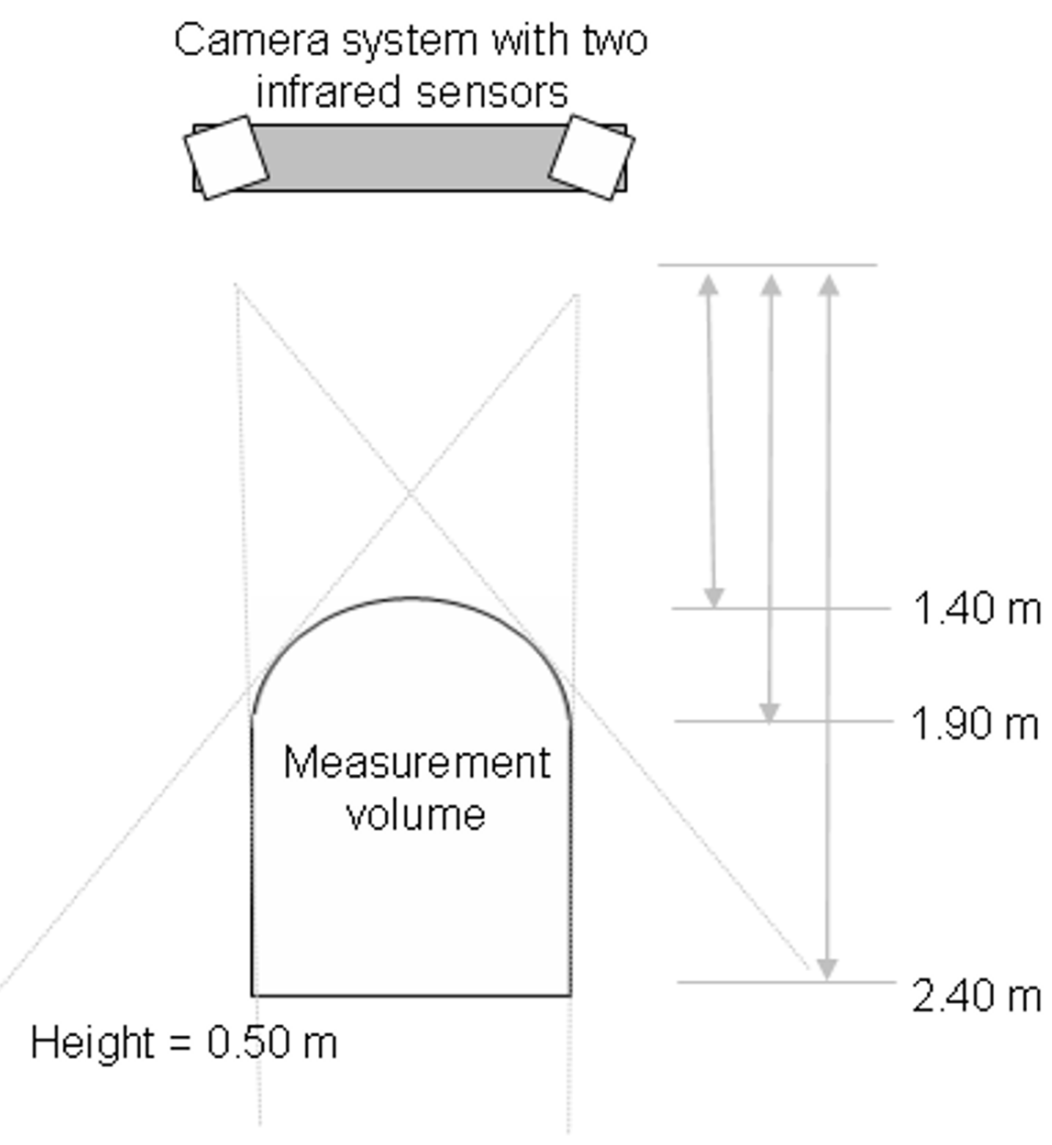
**Top view of the camera system, with the measurement volume as defined by the manufacturer**.

**Figure 4:**
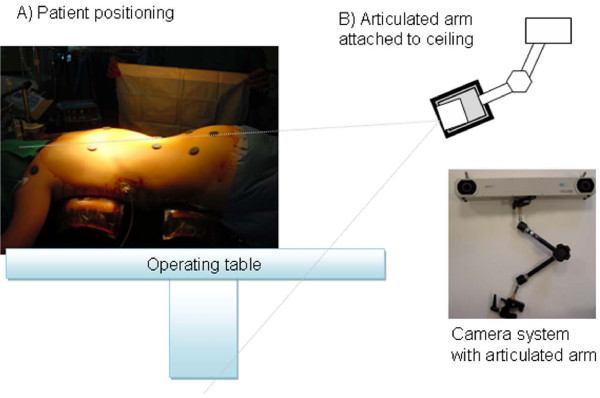
**Setup of the optoelectronic camera system in the operating room**. A) Patient positioning on the Relton-Hall frame and initial landmark identification using magnets. B) The camera is fixed on the ceiling using an articulated arm and oriented to provide the optimal coverage of the measurement volume over the patient's trunk.

Intraoperative motion of the trunk geometry is monitored using a Polaris infrared camera system, commonly used for surgical instrument tracking in orthopedic computer assisted surgery (Northern Digital, Waterloo, Canada). This camera system comprises two infrared sensors, mounted at equal distance from the center of the camera system, and slightly rotated with a small angle to cover optimally the measurement volume defined by the manufacturer (Figure 3 and Figure 4B). Each of these sensors emits infrared light toward the front of the camera system. The infrared signal is then reflected on the passive markers. The reflected signal is detected by both sensors and the 3-D position of each passive marker is established by active triangulation from the detected signal by both infrared sensors [12]. The accuracy of the camera system is evaluated by the manufacturer at 0.35 mm root mean square (RMS) distance. Benchmarking of the accuracy of the camera system was also internally assessed by acquiring 3-D data in a controlled environment, simulating the intraoperative setup. Internal validation of the accuracy of tracking was evaluated at 0.41 +/- 0.05 mm RMS, computed by acquiring several frames of all landmarks disposed on a dummy designed to simulate patient positioning.

**Figure 5:**
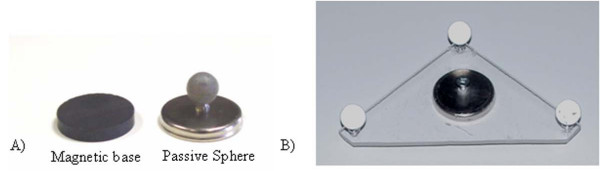
**Marker used for landmark tracking**. A) Mounting post, with magnetic base and passive markers reflecting the infrared light emitted by the camera. B) Triangular frame used for tracking of the pelvis and for defining a local reference frame for the measurements.

Prior to surgery, the patient is installed on the Relton-Hall frame and landmarks are identified using sterile magnetic base with double-faced tape (Figure 4A). The camera system is installed over the operating table using an articulated arm attached on the ceiling (Figure 4B) to allow continuous data collection without interfering with the surgical field. During this setup phase, position and orientation of the camera are optimized to ensure maximum coverage of the passive markers within the measurement volume of the camera system. After installation on the ceiling, the camera system remains fixed in this position during the entire surgery. The camera system is linked to a laptop computer, used to gather real time 3-D positions of the passive markers, and to display real-time clinical indices in 3-D. Nine custom-designed sterile mounting posts with passive markers (Figure 5A) are designed to fit the magnetic base over the draping sheets. The mounting post holds the passive markers using a metallic support composed of a straight vertical pin and a round metallic base. This mounting post is designed to fit over the draping sheets, clipped over the round magnetic base underneath. The mounting posts with their respective passive markers allow tracking of landmarks 1 to 9, as defined above. The landmark on the spinous process of S1 is used as a local reference frame for the measurements. Since a single point in 3-D cannot provide orientation information, three markers distributed on a triangular frame and mounted on the spinous process of S1 describe a reference plane, which is used to provide a robust representation of each axis relative to the patient and to track the displacement of S1 (Figure 5B). Each passive marker is installed on equidistant metallic supports fixed on a single acrylic triangular frame. A magnetic base is attached underneath the triangular frame to hold the marker over the draping sheet at S1.

The passive markers, their respective mounting posts and the triangular frame were sent to gas sterilization prior to surgery. After skin preparation and before installing the draping sheets, sterile magnetic bases are placed on each anatomical landmark defined on Figure 2A. Then, after installation of the draping sheets, individual mounting posts were clipped on corresponding magnetic bases, over the draping sheets. These markers correspond to landmarks 1 to 9. The triangular frame was fixed to landmark 10 and served as a reference to track the position and orientation of the spinous process of S1.

### External Trunk Geometry Tracking

**Figure 6:**
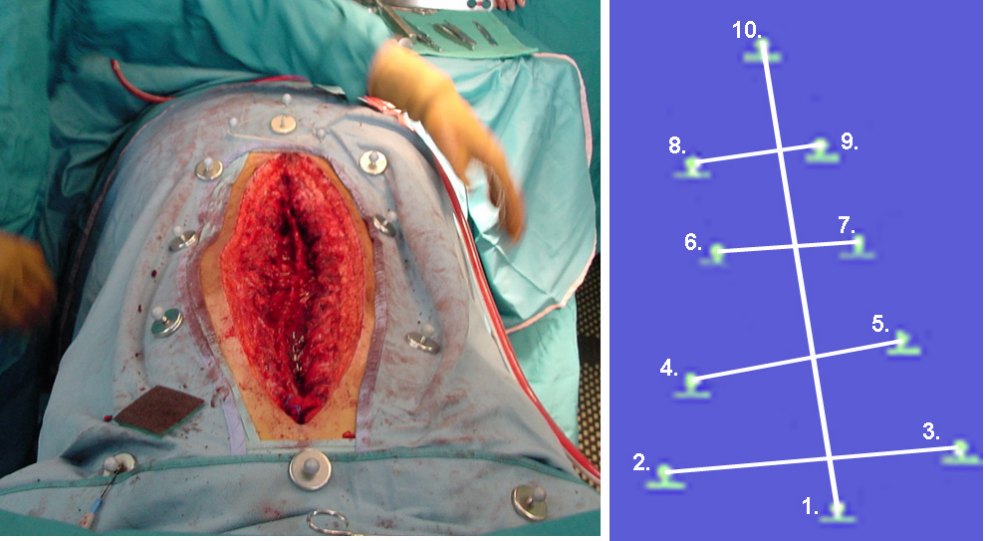
**A) View from the camera system, with the mounting post on landmark 1–9 to track the spine during and after instrumentation**. Landmark 10 is identified using the triangular frame. B) Visualization of the markers in 3-D using positions given by the camera system.

Continuous tracking for evaluation of the trunk geometry is performed during the surgery. Two sets of data acquisition were retained for each patient for the purpose of this study. A first data acquisition (stage I) was performed after insertion of the first rod before the rod rotation maneuver. Stage I therefore corresponds to the state prior to curve correction. A second data set (stage II) was acquired after fixation of both rods before wound closure, and is used to characterize the trunk geometry after final curve correction. 3-D positions were collected for a 10 second interval at 10 Hz for each stage. Since the patient is still breathing during continuous data acquisition, 3-D tracking was visually gated to select only the frames at the end of the respiratory cycle, to ensure a reproducible protocol across patients. Data collection was performed using 3-D visualization software that displays the position of all markers and a schematic representation of the trunk (Figure 6). The optical tracking system gathers 3-D position relative to a global reference frame relative to the camera fixed on the ceiling. The raw data, recorded in the axis system of the camera, are rotated and translated into the coordinate axis system defined by the Scoliosis Research Society (SRS) [13]. This reference frame is defined as follow: the X axis follows the gravity line, which is directed anteriorly with respect to the prone patient, and the Y- and Z-axes point toward left and cephalad directions, respectively. The origin of the axis system is at the spinous process of S1. The orientation of X, Y and Z axis from the origin is identified using the passive markers on the triangular frame defining a local reference frame, including which axis correspond to the left and cephalad directions. The initial orientation of the triangular frame, to ensure proper identification of the reference frame, is set by aligning two of the triangular frame's passive markers, with the markers on the pelvis (landmark 8–9) and this procedure is assisted using the software. The software also computes in real-time five geometric indices of the trunk based on the 3D position of the sensors in the SRS axis system (Figure 2B). A positive value for a linear index (in mm) is directed along the X-, Y- or Z-axis whereas a positive value for an angular index (in degrees) indicates a counterclockwise rotation with respect to an X-, Y- or Z- positive axis. The position of the markers as well as the trunk indices and the Cobb angles were compared between the two stages of surgery for each patient.

## Results

Primary Cobb angle measured on standing pre and post operative standing posterior-anterior radiographs are presented in Table 1 for all patients. Intra-operative tracking results are presented in Table 2, 3. Table 2 presents the absolute difference between the position of the landmarks during and after final correction of the spine. Differences are observed mainly for the shoulder landmarks. Slight relative displacement of the landmark at C7 is observed between both stages.

**Table 2:** Landmark (1–10) displacement recorded in mm during and after surgical correction of scoliosis

Landmarks	Patient 1 Displacement (mm)	Patient 2 Displacement (mm)	Patient 3 Displacement (mm)	Patient 4 Displacement (mm)	Patient 5 Displacement (mm)
1	1.39	0.23	1.18	0.65	0.15
2	12.20	9.40	5.60	10.05	5.63
3	2.45	1.28	6.98	10.66	8.45
4	5.72	2.09	4.63	8.01	8.40
5	1.64	3.11	5.47	9.93	4.82
6	8.58	2.16	2.76	5.28	6.35
7	1.00	0.24	2.85	7.53	1.58
8	3.78	1.51	1.92	3.79	2.30
9	0.75	0.56	1.97	6.95	0.63
10	0.00	0.00	0.00	0.00	0.00

**Table 3:** Trunk external indices computed during and after surgical correction of scoliosis. The indices showing improvement are outlined in bold.

	Patient 1		Patient 2		Patient 3		Patient 4		Patient 5	
Indices	Stage I	Stage II	Stage I	Stage II	Stage I	Stage II	Stage I	Stage II	Stage I	Stage II

Shoulders Orientation – X (deg)	**9.63**	**5.99**	14.58	15.74	**-6.77**	**-2.77**	-1.32	4.91	**5.33**	**-1.81**
Shoulders Orientation – Z (deg)	**-9.11**	**-1.86**	**3.39**	**1.60**	2.00	2.54	4.80	-7.83	-1.04	5.46
Balance – X (mm)	-1.29	-1.97	-5.20	-5.68	-0.11	-0.48	-10.72	-20.15	-0.58	2.24
Balance – Y (mm)	**-19.97**	**-19.80**	**28.00**	**22.58**	**-25.05**	**-23.12**	**-32.95**	**-22.89**	-41.34	-45.96
Spine length (mm)	51.11	52.49	53.72	53.46	48.64	49.82	38.28	37.63	50.53	50.68

**Figure 7:**
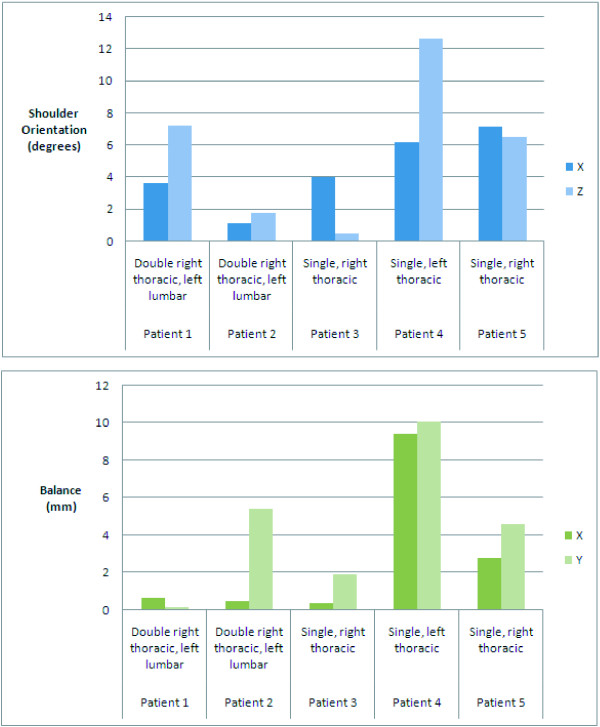
**Absolute difference of intraoperative trunk indices during and after surgical correction of scoliosis**.

Table 3 presents the measurements during and after surgical correction of the scoliosis. Results for patients 1, 3 and 5 shows improvement of the shoulder balance between both stages as well. Improvement of the global balance is observed for patients 1, 2, 3 and 4. At last, a minor increase of the spinal length can be noticed for patient 1, 3 and 5. Figure 7 illustrates the changes between stage I and II, in absolute values (without considering the direction of the changes). This result is obtained by performing the absolute value of the difference between the clinical measurement for stage II and stage I. Two patients with a double right thoracic, left lumbar curve presented absolute changes for the shoulder rotation between both stages. Although they presented a similar curve type, in the first case, the shoulder rotation is mainly affected by the final correction (shoulder absolute difference: X: 3.64 degrees and Z: 7.25). In the second double thoracic curve, shoulder rotation difference is observed, but it is mainly for the spinal balance (5.42 degrees) that major changes are observed. The single thoracic curve (Patient 3) presented shoulder rotation mainly along the X axis (4.00 degrees) between stage I and II. Clinical indices for the left thoracic curve showed the most obvious variations (shoulder absolute difference: X: 6.23 degrees and Z: 12.63). Balance in X and Z is also greatly affected for this patient (balance absolute difference: X: 9.43 degrees and Z: 10.06). The last remaining patient, with a single right thoracic curve, showed moderate to large shoulder rotation absolute difference (X: 7.14 degrees and Z: 6.5).

## Discussion

This study presents the next step from the study of [7] into developing an intra operative tool to monitor changes in external trunk geometry in real time during scoliosis surgery. This technique is appealing in several ways: 1) it provides 3-D data that can be correlated with the external shape of the patient during initial positioning on the operative table 2) it paves the way to provide continuous spatial tracking during the surgery without any interference or added radiation exposure to the patient, 3) it provides a simple and feasible workflow to be used for intraoperative setup, 4) It can provide direct real time feedback to the surgeon in order to evaluate the efficacy of his correction maneuvers, and 5) It could be used to compare the efficiency of different instrumentation systems or surgical maneuvers to correct scoliosis. In particular, in patients with significant global imbalance, pelvic or shoulder obliquity, the current technique could allow the surgeon to better assess these parameters during his correction maneuvers, so to decrease the use of intra-operative radiographs.

The optical sensors recorded only slight variations of rotations angles (5–10 degrees) on the Relton-Hall frame, where any displacement of the supports during the procedure is prohibited. It is known that about 30% of the correction is attributed by the positioning of the patient on the frame [14,15]. Accordingly, continuous tracking of the trunk geometry during the positioning step is advantageous since it would allow optimal positioning and curve correction before the surgery. Moreover, it can provide insight about the patient's external trunk geometry under draping sheets, which cannot be assessed from radiographs. This becomes extremely useful to provide quantitative information about the positioning of the patient on the Relton-Hall frame before, during and after surgery. Therefore, the technique described above could assist the surgeon in obtaining a global view of the patient's deformation over the surgical field during positioning stage and all along the surgery. Although only AIS patients were evaluated in this study, the technique could be applied to any patient with spinal deformity, such as patients with neuromuscular scoliosis who often present with severe imbalance and pelvic obliquity.

This study presents an accurate intra-operative system that could be used to assist spine surgeons to evaluate patient positioning and external trunk correction. The workflow described in this study involves only minor modifications to the current surgical setup. Moreover, this technique is not harmful to the patient and no radiations are involved for providing intraoperative guidance. This is appealing to obtain real-time continuous data in an intra operative setting. Improvements from the previous study involving magnetic tracking [7] are: 1) the optoelectronic camera system does not interfere in any way with the current surgical setup. 2) the tracking system does not require any wiring to connect the markers, since passive spheres do not require any electrical input to be tracked in 3-D. 3) the optoelectronic camera are not subject to inaccuracy due to ferromagnetic material, hence continuous data acquisition is feasible during the surgery.

Trunk geometry can sometimes be difficult to assess due to displacement and elasticity of the skin. Many authors, mainly targeted for gait analysis [17] and for spinal motion measurement [18], proposed external landmark tracking for real-time assessment of the trunk. In these cases, skin displacement might occur when the motion is relatively large, which was not the case in our study. Displacement on the skin could potentially alter the true location of a landmark used to compute an angle. However, in the current study, the landmarks that are used to compute angles are all distant to each other and an error of even 1 cm will affect the resulting angle by less than 2 degrees if the landmarks are 30 cm apart. Also, by considering only indices after exposure of the spine, prior to instrumentation, it is possible to reduce the errors due to skin displacement and to obtain a more robust trunk tracking. Moreover, the patient is sedated and supported by the surgical frame. Only the corrective motion by the clinician and the respiratory function might alter the positioning of the landmarks on the patient. To verify this hypothesis, a larger number of landmarks, could be disposed in a uniform distribution on the patient, to quantify skin displacement local to each landmarks.

## Conclusion

This study presented preliminary results of intraoperative trunk tracking using an opto- electronic camera system used commonly in computer assisted orthopedic surgery system. The workflow presented in this study can be mapped easily into existing computer assisted systems to provide online guidance while correcting spinal deformity, to achieve an optimal cosmetic correction of the trunk. However, the work presented in this preliminary study requires further investigations and clinical validation to indicate how measurements during surgery can accurately predict postoperative residual deformity or imbalance such as rib hump, shoulder imbalance, coronal and sagittal trunk imbalance in the standing (or sitting) position. For this purpose, comparisons between pre-, per- and and postoperative measurements will be the focus of future studies.

## Consent

Written patient consent was obtained for publication of the report.

## Competing interests

This research was conducted with the financial support of MENTOR, a strategic training program of the Canadian Institutes of Health Research.

## Authors' contributions

LD and JMMT were responsible for the clinical data collection. All authors were involved for the design of the study, as well as the data analysis. All authors participated in the preparation and approval of the final manuscript.
